# Transcriptomic Responses in the Livers and Jejunal Mucosa of Pigs under Different Feeding Frequencies

**DOI:** 10.3390/ani9090675

**Published:** 2019-09-12

**Authors:** He Zhang, Jiajun Liu, Xinpei Zhang, Jin Wang, Yong Su, Weiyun Zhu

**Affiliations:** Laboratory of Gastrointestinal Microbiology, Jiangsu Key Laboratory of Gastrointestinal Nutrition and Animal Health, College of Animal Science and Technology, Nanjing Agricultural University, Nanjing 210095, China

**Keywords:** feeding frequency, growth performance, jejunal mucosa, lipid metabolism, transcriptional profile

## Abstract

**Simple Summary:**

Nutrition management strategies are closely related to body development and health, and feeding frequency affects pig feed intake, feed efficiency, body composition, and growth performance. However, the effect of feeding one time daily and two times daily on the intestine has been given less attention. In this study, we investigated the transcriptomic responses induced in the livers and jejunal mucosa of growing pigs by daily feeding schedules. We found that when compared with feeding once daily, two times feeding had no significant effect on the growth performance of growing pigs with the same average daily feed intake. A two meals regimen reduced the concentration of triglycerides in serum and liver, affected the body metabolism by promoting lipid transport, lipogenesis, fatty acid oxidation, chylomicron formation and transport, gluconeogenesis, and inhibiting adipocyte differentiation. These findings support the idea that different feeding regimens could affect lipid metabolism and can be effective in nutritional strategies against metabolic dysfunction.

**Abstract:**

Feeding frequency in one day is thought to be associated with nutrient metabolism and the physical development of the body in both experimental animals and humans. The present study was conducted to investigate transcriptomic responses in the liver and jejunal mucosa of pigs to evaluate the effects of different feeding frequencies on the body’s metabolism. Twelve Duroc × Landrance × Yorkshire growing pigs with an average initial weight (IW) of 14.86 ± 0.20 kg were randomly assigned to two groups: feeding one time per day (M1) and feeding two times per day (M2); each group consisted of six replicates (pens), with one pig per pen. During the one-month experimental period, pigs in the M1 group were fed on an ad libitum basis at 8:00 am; and the M2 group was fed half of the standard feeding requirement at 8:00 am and adequate feed at 16:00 pm. The results showed that average daily feed intake, average daily gain, feed:gain, and the organ indices were not significantly different between the two groups (*p* > 0.05). The total cholesterol (T-CHO), triglyceride (TG), high-density lipoprotein-cholesterol (HDL-C), and low-density lipoprotein-cholesterol (LDL-C) concentrations in the serum, and the TG concentration in the liver in the M2 groups were significant lower than those in the M1 group, while the T-CHO concentration in the liver were significant higher in the M2 group (*p* < 0.05). Jejunal mucosa transcriptomic analysis showed the gene of *Niemann-Pick C1-Like 1 (NPC1L1)*, *Solute carrier family 27 member 4 (SLC27A4), Retinol binding protein 2 (RBP2), Lecithin retinol acyltransferase (LRAT), Apolipoprotein A (APOA 1, APOA 4, APOB,* and *APOC 3)* were upregulated in the M2 group, indicating that fat digestion was enhanced in the small intestine, whereas *Perilipin* (*PLIN1* and *PLIN2*) were downregulated, indicating that body fat was not deposited. *Fatty acid binding proteins (FABPs)* and *Acetyl-CoA acyltransferase 1 (ACAA1)* were upregulated in the M2 group, indicating that two times feeding daily could promote the oxidative decomposition of fatty acids. In conclusion, under the conditions in this study, the feeding frequency had no significant effect on the growth performance of pigs, but affected the body’s lipid metabolism, and the increase of feeding frequency promoted the fat digestion in the small intestine and the oxidative decomposition of fatty acids in the liver.

## 1. Introduction

It is well known that nutrition management strategies are closely related to growth performance and health, and the feeding frequency is one of the key factors affecting the growth of the body and feed efficiency [1,2]. Previous studies have reported that different feeding frequencies affect the feed intake, feed efficiency, body composition, and growth performance of pigs [2,3,4,5,6]; however, due to the different trial conditions, conflicting results associated with changes in feed efficiency and body composition have been reported in previous studies [2,4]. Feeding once or twice per day are common feeding patterns used in the swine production of China, therefore, it is necessary to investigate and compare the responses on growth performance and the body metabolism of pigs under these two feeding patterns.

So far, the regulation mechanism of feeding frequency on the growth and body metabolism of pigs is not fully clear. Higher feeding frequency could improve the glucose clearance rate and prevent ruminants from accumulating fat in visceral adipose depots due to higher insulin sensitivity [7]. Meal frequency could change the time-course profiles of plasma concentrations of glucose, insulin, and lactate in pig [2]. A twelve meals regimen increased the liver concentration of total lipid, glycogen, and triglyceride than that in the two meals daily pigs [8]. Compared with pigs fed ad libitum, the activities of citrate synthase, β-hydroxylacyl-CoA dehydrogenase, and lactate dehydrogenase were greater in the longissimus muscle of the two meals daily regimen [9]. The intestine and liver are the major digestive, absorption, and metabolism organs of the body and play a key role in the metabolism of nutrients [10,11]. To date, information on the transcriptomic responses in the liver and jejunal mucosa of pigs under different feeding frequencies is not available.

The present study hypothesized that compared with feeding once daily, pigs feeding twice daily could regulate the gene expression of the intestine and liver, eventually affecting the body metabolism. Therefore, the objective of this study was to compare the growth performance, body metabolism, and transcriptional profiles in the livers and jejunal mucosa of pigs under different feeding frequencies.

## 2. Materials and Methods

### 2.1. Experimental Animals, Design, and Diet

This experiment was approved and conducted under the supervision of the Animal Care and Use Committee of Nanjing Agricultural University (Nanjing, Jiangsu Province, China). The ethic code is SYXK (SU) 2017–0007. All pigs were raised and maintained on a local commercial farm under the care of the Animal Care and Use Guidelines of Nanjing Agricultural University. In our study, all animals were individually housed in metal-floor cages (height, 0.85 m; length, 1.2 m; width, 0.70 m) with a smooth walled pan, a nipple drinker, and a feeder. The room temperature was maintained at 25 ± 2 °C during the experimental period. All pigs were kept at a 24 h light–dark cycle, with lights being turned on from 8:00 am to 20:00 pm. Twelve 42-d Duroc × Landrance × Yorkshire growing barrows (BW = 14.86 ± 0.20 kg) were randomly allocated to the one time feeding daily (M1) group and two times feeding daily (M2) group, and each group consisted of six replicates (pens) with one pig per pen. Pigs in the M1 group were fed the adequate diet on an ad libitum basis at 8:00 am on each experimental day. Pigs in the M2 group were fed half of the standard feeding requirement according to the National Research Council (NRC, 2012) at 8:00 am and adequate feed at 16:00 pm [12], and the refusals were removed from the feeder and weighed at 20:00 pm. The composition and nutrient level of the diet in our study is shown in Table 1, and all pigs had free access to water during the 30-day trial period. Individual initial and final body weight as well as feed intake were registered during the experiment to calculate the average daily gain (ADG) and average daily feed intake (ADFI). Feed efficiency (feed:gain) was expressed as F:G.

### 2.2. Sampling

After 12 h fasting, all pigs were euthanized at 8:00 am with a jugular vein injection of 4% sodium pentobarbital solution (40 mg/kg BW). Blood samples were collected and centrifuged at 2000× g for 10 min at 4 °C, and the supernatant stored at −70 °C until subsequent biochemical analysis. The animals were bled and opened immediately, and the liver, kidney, spleen, heart, and the entire gastrointestinal tract were removed and weighed. Tissues from the liver and jejunal mucosa were collected and kept in liquid nitrogen, then stored at −80 °C for further transcriptome analysis.

### 2.3. Biochemical Parameters of Serum and Liver

Glucose, total bile acid (TBA), triglyceride (TG), total cholesterol (T-CHO), high-density lipoprotein-cholesterol (HDL-C), and low-density lipoprotein-cholesterol (LDL-C) in the serum of pigs were measured with an Olympus AU400 Automatic Biochemical Analyzer (Olympus Optical Co., ltd., Tokyo, Japan). The concentrations of liver T-CHO, TG, HDL-C, LDL-C, and TBA were determined by using commercial biochemical assay kits (Nanjing Jiancheng Bioengineering Institution, Nanjing, China).

### 2.4. Library Construction for RNA Sequencing and Sequencing Procedures

Total RNA of the liver tissues and jejunal mucosa were isolated using an RNeasy mini kit (Qiagen, Hilden, North Rhine-Westphalia, Germany). As there were six replicates in each group, three biological replicates were randomly selected for the RNA-Seq to reduce the costs of the experiment. A total amount of 1 μg RNA per sample was used as input material for the RNA sample preparations. Sequencing libraries were generated using the NEBNext UltraTM RNA Library Prep Kit for Illumina (New England BioLabs, Ipswich, MA, USA) following the manufacturer’s recommendations, and index codes were added to attribute sequences to each sample. In order to select cDNA fragments of preferentially 240 bp in length, the library fragments were purified with AMPure XP system (Beckman Coulter, Beverly, MA, USA). Then, 3 μL USER Enzyme (New England BioLabs, Ipswich, MA, USA) was used with size-selected, adaptor-ligated cDNA at 37 °C for 15 min followed by 5 min at 95 °C before PCR. Then PCR was performed with Phusion High-Fidelity DNA polymerase (New England BioLabs, Ipswich, MA, USA), universal PCR primers, and index (X) Primer (New England BioLabs, Ipswich, MA, USA). Finally, the PCR products were purified (AMPure XP system) and the library quality was assessed on an Agilent Bioanalyzer 2100 system (Agilent Technologies, Santa Clara, CA, USA). The clustering of the index-coded samples was performed on a cBot Cluster Generation System using the TruSeq PE Cluster Kit v4-cBot-HS (Illumia (Illumina, San Diego, CA, USA) according to the manufacturer’s instructions. After cluster generation, the library preparations were sequenced on an Illumina platform and paired-end reads were generated.

### 2.5. Quantitative Real-Time PCR

The first-strand cDNA synthesis was performed using 1 μg of total RNA with a reverse transcription kit (Takara Bio, Shiga, Japan). The expression of genes *Apolipoprotein A1 (APOA1), Apolipoprotein A4 (APOA4), Apolipoprotein C3 (APOC3), Niemann-Pick C1-Like 1 (NPC1L1), Acetyl-CoA acyltransferase 1 (ACAA1), Fatty acid binding protein 1 (FABP1)*, and *Phosphoenolpyruvate carboxykinase 1 (PCK1)* were measured by quantitative real-time PCR using an ABI 7300 sequence detector (SDS, Foster City, CA, US). The PCR reactions were performed in a final volume of 20 μL with the Roche SYBR Green PCR Kit (Roche, Hercules, CA, USA), according to the manufacturer’s instructions. The sequences of the primers are listed in Appendix A Table A1. The expression of the genes was calculated relative to the expression of *β-actin* with the formula 2^−ΔΔCt^ [13].

### 2.6. Statistics

Growth performance, organ weight, serum metabolites, and hepatic metabolite data were analyzed by SPSS version 22.0 software (SPSS Inc., Chicago, IL, USA) as a randomized complete block design. The pen was used as the experimental unit (n = 6), and the significance of differences between the M1 and M2 groups was evaluated by a Student’s *t* test. Significant differences were declared when *p* < 0.05.

Genes with altered expression (FC > 1.5 or < 0.67; *p* < 0.05) were selected for further study. Additionally, the genes identified between the two groups were mapped to the Gene Ontology (GO, http://www.geneontology.org/, accessed 16 July 2018) terms and Kyoto Encyclopedia of Genes and Genomes (KEGG, http://www.genome.jp/kegg/, accessed on 16 July 2018) pathways to identify potential pathways associated with dietary treatment. GO enrichment analysis of the differentially expressed genes (DEGs) was implemented based Wallenius non-central hyper-geometric distribution [14]. KOBAS [15] software (http://kobas.cbi.pku.edu.cn/) was used to test the statistical enrichment of the DEGs in the KEGG pathways. ClueGO (version 2.5.4) and CluePedia (version 1.5.4), a plugin for Cytoscape version 3.7.1 (NIGMS, Bethesda, MD, USA), were used for functionally grouped network analysis [16,17].

## 3. Results

### 3.1. Growth Performance

All pigs were kept healthy during the experiment. No differences in final body weight (FBW), average daily gain (ADG), average daily feed intake (ADFI) and feed:gain (F:G) were found between the two groups (Table 2). The weight of the liver, kidney, spleen, whole intestinal tract, and their percentage of body weight were not affected by feeding frequency (Appendix A Table A2).

### 3.2. Serum and Liver Metabolites

As shown in Table 3, concentrations of serum T-CHO, TG, HDL-C, and LDL-C of pigs in the M2 group were lower than in the M1 group (*p* > 0.05), while no significant differences in the concentrations of serum glucose and TBA were found between the two groups. The concentrations of liver TBA, HDL-C, and LDL-C were not affected by feeding frequency. The twice feeding regimen significantly decreased the concentration of liver TG, while significantly increased the concentration of liver T-CHO (*p* < 0.05).

### 3.3. Gene Expression Profiles in the Liver and Jejunal Mucosa

The gene expression profiles of the liver showed that 256 differentially expressed transcripts were identified and annotated between the two groups at the particular cutoff criteria (FC ≥ 1.5 or < 0.67; *p* < 0.05). In total, 145 genes were upregulated and 111 genes were downregulated among the annotated genes. Overall, the most upregulated gene was *TNF receptor superfamily member 17 (TNFRSF 17)*, which showed a 33.09-fold increase with the free feeding treatment, whereas the most downregulated gene was *Wnt-11*, with a 8.82-fold decrease.

In the jejunal mucosa, 817 differentially expressed transcripts were identified and annotated between the two groups at the particular cutoff criteria (FC ≥ 1.5 or < 0.67; *p* < 0.05). In total, 401 genes were upregulated and 416 genes were downregulated among the annotated genes. Overall, the most upregulated gene was *fibroblast growth factor 19 (FGF19)*, which showed a 6.34-fold increase with the free feeding treatment, whereas the most downregulated gene was *homeobox B3 (HOXB3)*, with a 9.25-fold decrease.

**Figure 1:**
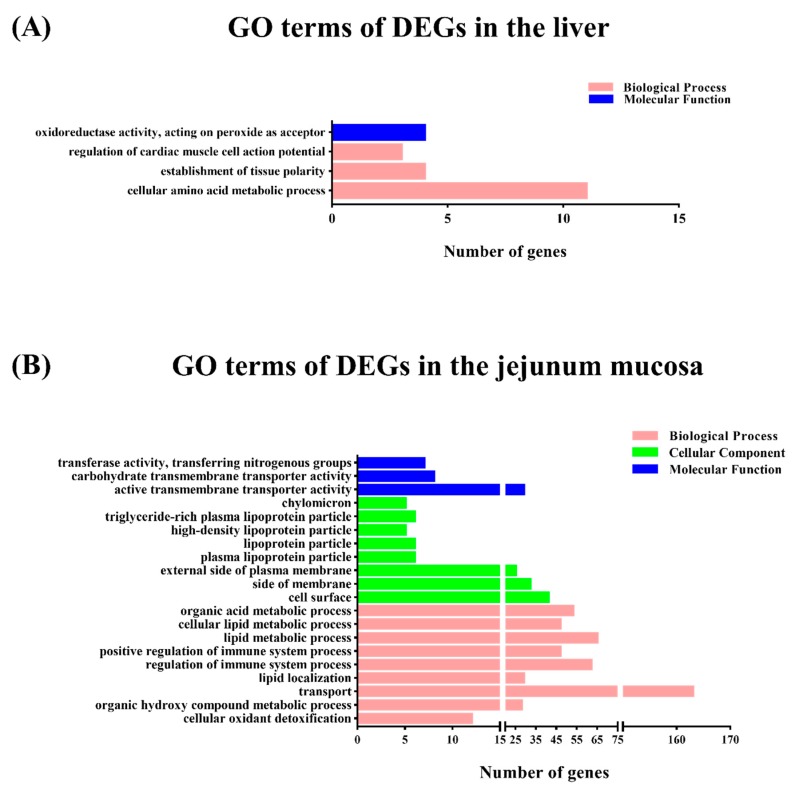
Function classifications of Gene Ontology (GO) terms of differential expressed transcripts between the one meal per day (M1) and two meals per day (M2) groups. A *p* value < 0.05 and fold change (FC) ≥ 1.5 were used as thresholds to select significant GO categories. The green bars indicate the number of differentially expressed genes (DEGs) enriched in molecular function, the blue bars indicate the number of DEGs enriched in cellular component, and the pink bars indicate the number of DEGs in the biological process. (**A**) Analysis of the GO terms of DEGs in the liver. (**B**) Analysis of the GO terms of DEGs in the jejunal mucosa.

The DEGs were subjected to GO functional category analysis and then by GO enrichment analysis. All DEGs were enriched in the three main functional categories including biological process, cellular components, and molecular function (FC ≥ 1.5 or < 0.67; *p* < 0.05). In the biological process level of the GO categories, most of the genes in the liver were significantly represented in the cellular amino acid metabolic process, establishment of tissue polarity, and regulation of cardiac muscle cell action potential (Figure 1A). In the jejunal mucosa, most of the genes were significantly represented in the lipid metabolic process, regulation of immune system process, organic acid metabolic process, positive regulation of immune system process, cellular lipid metabolic process, small molecule biosynthetic process, and so forth (Figure 1B).

**Figure 2:**
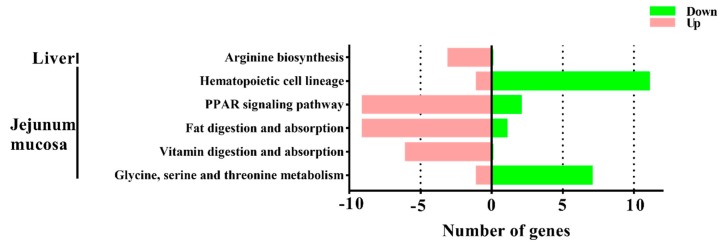
Pathway assignments of differential expressed transcripts in the liver and jejunal mucosa of pigs based on the Kyoto Encyclopedia of Genes and Genomes (KEGG). ClueGO (a plugin for Cytoscape) was used for KEGG analysis, and a *p* value < 0.05 was used as the threshold to select significant KEGG pathways. The pink bars indicate the number of upregulated genes, while the green bars indicate the number of downregulated genes.

**Figure 3:**
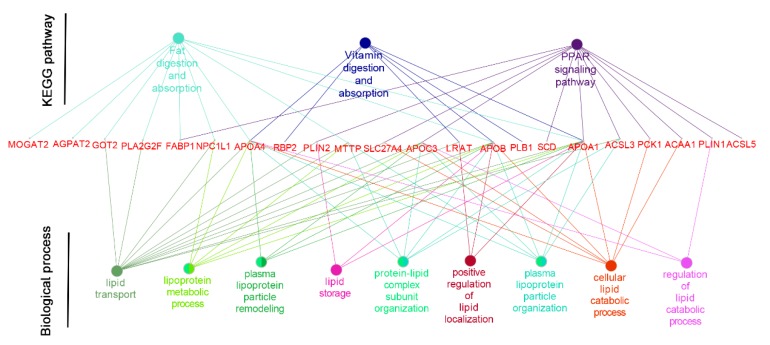
Genetic network and functions of PSGs (positively selected genes) related with the KEGG pathway of the PPAR signaling pathway, fat digestion and absorption, vitamin digestion and absorption. Functions were defined using Gene Ontology (GO) and the Kyoto Encyclopedia of Genes and Genomes (KEGG) annotations and the network was constructed by ClueGO and the CluePeidia plugin in Cytoscape. Each solid circle represents a gene or a functional category, the colored line show ontology relations. *APOA1: Apolipoprotein A1; APOA4: Apolipoprotein A4; APOB: Apolipoprotein B; AGPAT2: 1-acylglycerol-3-phosphate O-acyltransferase 2; MTTP: microsomal triglyceride transfer protein; MOGAT2: monoacylglycerol O-acyltransferase 2; NPC1L1: Niemann-Pick C1-Like 1; FABP1: Fatty acid binding protein 1; GOT2: glutamic-oxaloacetic transaminase 2; SCD: stearoyl-CoA desaturase; APOC3: Apolipoprotein C3; SLC27A4: solute carrier family 27 member 4; ACSL3: acyl-CoA synthetase long chain family member 3; ACSL5: acyl-CoA synthetase long chain family member 5; ACAA1: Acetyl-CoA acyltransferase 1; PCK1: Phosphoenolpyruvate carboxykinase 1; PLIN1: Perilipin 1; PLIN2: Perilipin 2; PLA2G2F: phospholipase A2 group IIF;* and *PLB1: phospholipase B1.*

**Figure 4:**
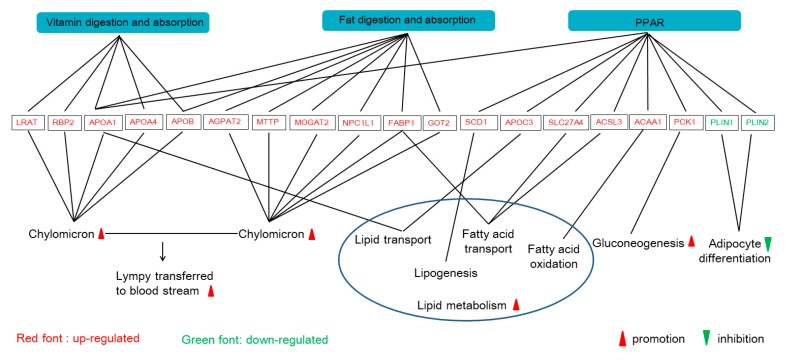
The signaling network of the gene and biological functions caused by the two times feeding regimen. The relationships between the genes and biological function are indicated by the lines. *LRAT: Lecithin retinol acyltransferase; RBP2: Retinol binding protein 2; APOA1: Apolipoprotein A1; APOA4: Apolipoprotein A4; APOB: Apolipoprotein B; AGPAT2: 1-acylglycerol-3-phosphate O-acyltransferase 2; MTTP: microsomal triglyceride transfer protein; MOGAT2: monoacylglycerol O-acyltransferase 2; NPC1L1: Niemann-Pick C1-Like 1; FABP1: Fatty acid binding protein 1; GOT2: glutamic-oxaloacetic transaminase 2; SCD: stearoyl-CoA desaturase; APOC3: Apolipoprotein C3; SLC27A4: solute carrier family 27 member 4; ACSL3: acyl-CoA synthetase long chain family member 3; ACAA1: Acetyl-CoA acyltransferase 1; PCK1: Phosphoenolpyruvate carboxykinase 1; PLIN1: Perilipin 1;* and *PLIN2: Perilipin 2.*

To better understand the functional changes, the DEGs were subjected to the KEGG database (*Sus scrofa*) for pathway enrichment analysis. The significantly changed pathway in the liver of pigs was arginine biosynthesis. In the jejunal mucosa, the significant enriched pathways included glycine, serine and threonine metabolism, the peroxisome proliferators-activated receptor (PPAR) signaling pathway, fat digestion and absorption, vitamin digestion and absorption, hematopoietic cell lineage (Figure 2, gene information is shown in Table 4). DEGs in the PPAR signaling pathway, fat digestion and absorption, vitamin digestion and absorption were constructed in a co-expression network by using the ClueGO and CluePeidia plugin in the Cytoscape network analyzer tool (Figure 3). The genes of *NPC1L1*, *GOT2*, *FEBP1*, *SLC27A4*, *ACSL3*, *ACSL5*, *MTTP*, *ACAA1*, *PCK1*, *APOA1*, *APOA4*, *APOB*, *PLN1*, and *PLN2* may play an important role in the network. The related pathways containing these selected genes were analyzed for further research (Figure 4). In detail, several genes involved in the formation of chylomicron (*LRAT*, *RBP2*, *APOA1*, *APOA4*, *APOB*, *AGPAT2*, *MTTP*, *MOGAT2*, *NPC1L1*, *FABP1*, and *GOT2*), lipid metabolism (*APOA1*, *APOC3*, *FABP1*, *SLC27A4*, *ACSL3*, *SCD1*, and *ACAA1*), and gluconeogenesis (*PCK1*) were upregulated, and several genes involved in adipocyte differentiation (*PLIN1*, *PLIN2*) were downregulated.

### 3.4. Validation of RNA-Seq Results by qRT-PCR

To validate the transcriptomic results by quantitative RT-PCR (qRT-PCR), seven upregulated genes (*FABP1*, *ACAA1*, *NPC1L1*, *PCK1, APOA1*, *APOA4*, and *APOC3*) were validated. As shown in Table 5, the results showed that the expression profiles of these genes detected by qRT-PCR were consistent with those detected by transcriptome, which confirmed the reliability of our RNA sequencing data.

## 4. Discussion

Restricted meal frequency is considered a potentially effective treatment for metabolic disease besides limited caloric intake [18,19,20]. A previous study showed that the liver fat content of pigs fed two meals daily was lower than that in pigs with twelve meals [8]. In the present study, compared with the M1 group, the M2 regimen significantly decreased the concentrations of serum T-CHO, TG, HDL-C, LDL-C, and liver TG, although the growth performance of pigs was not affected. In order to explore the underlying mechanisms, transcriptomic responses in the livers and jejunal mucosa of growing pigs were investigated following two different feeding frequency regimens.

By recording the feeding intake at different time points in one day, we found that pigs in the M1 group consumed 75–85% of the total diet before 16:00 pm every day, while the M2 group only consumed half of the total diet at 16:00 pm every day, which indicates that the feeding patterns impacted the meal regimen. However, across the one-month trial, the two different feeding regimens had no significant effect on the growth performance of the growing pig. The average daily feed intake (ADFI) was correlated with the time of eating, therefore, no difference in ADFI in our study was likely due to the same duration of eating time in the M2 group when compared to free access. Colpoys et al. observed a decrease in ADFI in gilts fed two meals daily when compared to free access [5]. Newman et al. observed a tendency for boars fed two 1-h meals to eat less than ad libitum [4]; however, the ADFI was not significantly changed when boars were fed two 90-min meals. The inconsistent results may partly be due to the differences in the sex of the pigs used in different studies. In addition, the different growth period of pigs used in this study may partly explain the difference between earlier studies [2,3,4]. Schneider et al. pointed out that feeding two or six meals daily had the same effect on the growth performance of gestating gilts [21], but the average daily gain (ADG) of gilts was increased from day 0 to 42. In pigs maintained on a high fat dietary regimen, two meals per day decreased the fat deposition content when compared to twelve meals per day [22]. However, Hatori et al. showed that an increase in the feeding frequency significantly increased the ADG of rats fed a normal diet or high-fat diet [18]. This suggests that different feeding regimens may have different effects on the growth performance of animals from different species, different genders, and different physiological stages. Both the diet composition and frequency of daily feeding could affect growth performance. 

The accumulation of triglyceride-rich lipoproteins (TRLs) in blood is believed to be related to the occurrence of atherosclerotic dyslipidemia. Blood TG level and/or the remnant-cholesterol (remnant-cholesterol = T-CHO – LDL-C – HDL-C) reflect the level of TRL [23]. In our study, the two times feeding regimen significantly decreased the concentrations of serum T-CHO, HDL-C, and LDL-C. In addition, pigs on the M2 regimen showed a decreased TG concentration in the serum and liver, which is consistent with the results of previous studies on pigs or rodents [2,18,19]. Our study supports the idea that different feeding regimens can affect lipid metabolism.

To gain insights into the mechanisms underlying this process, transcriptomic analysis was conducted to identify the DEGs between the M1 and M2 regimens. In the liver, only the arginine biosynthesis pathway was enriched by the M2 regimen, however, in the jejunal mucosa, the significant enriched pathways included glycine, serine, and threonine metabolism, the PPAR signaling pathway, fat digestion and absorption, vitamin digestion and absorption, and hematopoietic cell lineage, which suggests that the different feeding frequencies in our experiment appeared to have a more significant effect on bay metabolism at the intestinal level.

The NPC1L1 transmembrane protein in the intestinal villi is responsible for the efficient and specific transport of cholesterol into the absorbing cells. Previous studies showed that *NPC1L1* gene ablation reduced the absorption of cholesterol by the small intestine, and increased cholesterol synthesis in the liver [24,25,26]. A previous study found that the concentration of cholesterol was significantly increased in transgenic mice for a human *NPC1L1* gene [27], while *NPC1L1*-deficient mice exhibited a drastic reduction of dietary cholesterol absorption [28,29]. The *GOT2* gene, known as plasma membrane-associated fatty acid-binding protein (FABPpm), can promote the transport and metabolism of fatty acids. It was reported that FABPpm participated in fatty acid metabolism by transporting long-chain fatty acids into the cell [30]. Acetyl-CoA acetyltransferase 2 (ACAT2) can catalyze the absorption of cholesterol absorbed in cells to form cholesteryl esters [31]. ACAT2-deficient in the intestine reduced the efficiency of the absorption and transportation of cholesterol by chyle particles and the absorption of cholesterol in the diet [32]. Under the action of apolipoprotein APOB48 and MTTP, cholesteryl esters were assembled together with triglycerides, phospholipids, and a small portion of free cholesterol to form chylomicrons, which could be secreted into lymphatic circulation through basement membrane [33,34,35]. In the present study, genes *NPC1L1*, *FABP1*, *MOGAT2*, and apolipoprotein (*APOA1, APOA4,* and *APOB*) were upregulated by the M2 regimen in fat digestion and absorption pathway, combined with the upregulation of genes *PLB1*, *RBP2*, and apolipoprotein (*APOA1, APOA4,* and *APOB*) in the vitamin digestion and absorption pathway, which indicates that the M2 regimen promoted the formation and transport of chylomicrons into the bloodstream, and the digestion of fat in the small intestine (Figure 4).

Long-chain acyl-coenzyme a synthase (ACSL) catalyzes the first step in the activation of intracellular fatty acid metabolism by converting long-chain fatty acids into long-chain acyl-CoAs [36]. The *ACSL3* gene can promote the synthesis of lecithin and the formation of intracellular lipid droplets, participate in fatty acid oxidation, and maintain lipid droplet formation in two metabolic pathways [37]. ACSL5 is a key enzyme in the process of fatty acid beta oxidation and triglyceride synthesis and metabolism in the animal body. Cao et al. upregulated the transcription of *ACSL3* gene by using cytokine tumor suppressor in HepG2 cells, enhanced β-oxidation, and reduced the TG content of cells [38]. Acetyl-CoA acyltransferase (ACAA), also known as 3-Ketoacyl-CoA thiolase, includes two subtypes, ACAA1 and ACAA2, which catalyze the last step of fatty acid beta oxidation. Previous studies have shown that the increase of ACAA activity promotes the oxidation of fatty acids [39,40]. The *ACAA1* gene is mainly involved in physiological and biochemical processes such as the oxidation of very long-chain fatty acids, bile acid metabolism, and regulation of peroxisome proliferation [41]. In the present study, we found that the M2 regimen significantly upregulated the expression of genes *ACSL3* and *ACSL5* in pig jejunum, which suggests that two times feeding daily could increase fatty acid β-oxidation (Figure 4).

The *FABP1 (L-FABP)* could be affected by PPAR and in turn affect PPARα [42]. *FABP* can assist in the transport of fatty acids to the mitochondria into the β-oxidation pathway and transport to the endoplasmic reticulum to enter the triglyceride esterification pathway. A previous study on mice fed a high-fat diet indicated that the *L-FABP* gene promoted long-chain fatty acid oxidation and inhibited weight gain and obesity [43]. The over-expression of *L-FABP* could significantly increase the intake of long-chain fatty acid oxygen and medium-chain fatty acid in the nucleus [44]. When L-FABP binds to PPARα, it can enhance the transcriptional activity of PPAR to long-chain fatty acid oxidase, regulate the utilization and metabolism of intracellular fatty acid, and maintain the relative balance of fatty acid metabolism in vivo [44]. In adipocytes, Perilipin (PLIN) anchors on the surface of the lipid droplets to produce a barrier effect, which blocks TG from lipohydrolase [45,46]. In the present study, the *PLIN* gene was downregulated, and its barrier effect was weakened with the increase of lipohydrolase activity, then lipid droplets became significantly smaller, which accelerated fat decomposition and reduced paper deposition in cells. In addition, phosphoenolpyruvate carboxykinase (PCK), a rate-limiting enzyme of hepatic glycogenesis, was also upregulated in the M2 group. Therefore, the results suggest that the two times feeding regimen can increase fatty acid β-oxidation and reduce fatty acid accumulation (Figure 4). However, Liu et al. found that the protein abundance of PCK2 in glucose and energy metabolism, the protein abundance of APOA1, APOB, FABP1, MTTP, and GOT2 in the lipid metabolism of pigs with two meals a day were significantly higher than that of pigs with twelve meals a day [8]. The inconsistent results between the two studies may be partly due to the different feeding regimens and physiological stages of the pigs.

Interestingly, the transcriptome date of this study revealed significant changes in the arginine biosynthesis pathway in the livers of pigs. In the enriched arginine biosynthesis pathway, *ARG2* and *NAGS* genes in the M2 group were upregulated when compared with the M1 group. These genes contribute to the synthesis of glutamic acid, glutamine (glutamate), and proline in the small intestine of pigs, which are precursors for the synthesis of citrulline and arginine [47]. Arginine can detoxify through the urea cycle, promote insulin and growth hormone secretion, and regulate nutrient metabolism, and has been widely used as an immune nutrient in clinical practice [48]. However, there was no difference in the growth performance between the two groups, and the influence of feeding frequency on the immune function needs further study. A study of proteomics in the liver revealed 35 differentially expressed proteins in the liver between pigs fed two and twelve meals per day, which were involved in the regulation of glucose and energy metabolism, lipid metabolism, protein and amino acid metabolism, and other general biological process [8]. The inconsistent results between the two studies may be partly due to the different feeding frequency and diet composition used in the pig trials. In addition, the present study mainly compared the body response to different feeding frequencies at the transcriptomic level, therefore, the enzyme activity or protein expression are needed to be determined in further study.

## 5. Conclusions

The present study investigated the transcriptomic responses induced in the livers and jejunal mucosa of growing pigs by different feeding frequencies. We found that when compared with one time feeding daily, the two times feeding regimen had no significant effect on the growth performance of growing pigs with the same average daily feed intake. The two times feeding regimen reduced the concentration of triglycerides in the serum and liver, affected the body metabolism by promoting lipid transport, lipogenesis, fatty acid oxidation, chylomicron formation and transport, gluconeogenesis, and inhibited the adipocyte differentiation. These findings support the idea that different feeding regimens affect lipid metabolism and can be effective as nutritional strategies to prevent metabolic dysfunction.

## Figures and Tables

**Table 1:** Composition and nutrient level of the diet (air-dry basis).

Ingredient	Percentage (%)	Nutritional Compositions (%)	
Corn	70.0	Digestive energy (MJ/kg)	14.60
Soybean meal	18.0	Crude protein	16.00
Wheat bran	6.50	Lysine	1.23
Soybean oil	1.90	Methionine + Cystine	0.70
Lysine	0.69	Threonine	0.79
Methionine	0.24	Tryptophan	0.22
Threonine	0.30		
Tryptophan	0.07		
Calcium hydrogen phosphate	0.45		
Stone powder	0.50		
Salt	0.30		
Multivitamins ^1^	0.03		
Minerals ^2^	0.20		
Choline chloride (50%)	0.12		
Zeolite powder	0.60		
Antioxidant	0.05		
Antifungal agent	0.05		
Total	100.00		

^1^ The mineral supply per kg diet was as follows: Fe 165 mg, Zn 165 mg, Cu 16.5 mg, Mn 30 mg, Co 0.15 mg, I 0.25 mg, and Se 0.25 mg. ^2^ The multivitamin supply per kg diet was as follows: VA 11,000 IU, VD3 1000 IU, VE 16 IU, VK1 1 mg, VB1 0.6 mg, VB2 0.6 mg, d-pantothenic acid 6 mg, nicotinic acid 10 mg, VB12 0.03 mg, folic acid 0.8 mg, and VB6 1.5 mg.

**Table 2:** Effects of feeding frequency on body weight and feed intake of growing pigs ^1^.

Items	M1	M2	*p* Value
IW/kg	14.81 ± 0.34	14.90 ± 0.31	0.858
FBW/kg	38.58 ± 0.80	38.83 ± 1.02	0.833
ADG/kg	0.679 ± 0.020	0.684 ± 0.025	0.886
ADFI/kg	1.370 ± 0.004	1.360 ± 0.008	0.396
F:G	2.027 ± 0.063	1.945 ± 0.041	0.182

^1^ Data are presented as group mean ± SEM, n = 6. IW: initial weight; FBW: Final body weight; ADG: average daily gain; ADFI: average daily feed intake; F:G: feed:gain. M1: feeding once per day; M2: feeding twice per day.

**Table 3:** Effects of feeding frequency on serum metabolites and liver metabolites of growing pigs ^1^.

Organ	Items	M1	M2	*p* Value
Serum	TBA (μmol/L)	21.80 ± 2.328	41.25 ± 7.128	0.067
	Glucose (mmol/L)	7.720 ± 0.475	8.384 ± 0.589	0.406
	T-CHO (mmol/L)	2.507 ± 0.077	2.038 ± 0.071	0.001
	TG (mmol/L)	0.712 ± 0.046	0.518 ± 0.020	0.003
	HDL-C (mmol/L)	1.105 ± 0.054	0.808 ± 0.036	0.001
	LDL-C (mmol/L)	1.262 ± 0.030	1.106 ± 0.026	0.005
liver	TBA (μmol/g protein)	3.589 ± 0.442	3.147 ± 0.347	0.463
	T-CHO (mmol/g protein)	0.009 ± 0.001	0.064 ± 0.006	0.011
	TG (mmol/g protein)	0.051± 0.005	0.030 ± 0.004	0.016
	HDL-C (mmol/g protein)	0.556 ± 0.057	0.796 ± 0.082	0.180
	LDL-C (mmol/g protein)	0.014 ± 0.002	0.011 ± 0.001	0.170

^1^ Data are presented as group mean ± SEM, n = 6. M1: feeding once per day; M2: feeding twice per day. TBA: Total bile acid; T-CHO: Total cholesterol; TG: Triglycerides; HDL-C: high-density lipoprotein-cholesterol; LDL-C: low-density lipoprotein-cholesterol.

**Table 4:** Pathways enriched with differentially expressed genes in the livers and jejunum mucosa of pigs induced by feeding one time daily when compared with feeding twice daily ^1^.

Organ	KEGG Category	KEGG Name	GO Term	Gene	Change	% Associated Genes	*p* Value	*q* Value
jejunal mucosa	Metabolism	Amino acid metabolism	Glycine, serine, and threonine metabolism	*ALAS2*	Down	20.51	<0.0001	0.024
				*BPGM*	Down			
				*GATM*	Down			
				*GLDC*	Down			
				*GLYCTK*	Up			
				*PGAM2*	Down			
				*PHGDH*	Down			
				*PSAT1*	Down			
	Organismal Systems	Digestive system	Vitamin digestion and absorption	*RBP2*	Up	27.27	<0.0001	0.041
				*PLB1*	Up			
				*APOA1*	Up			
				*APOA4*	Up			
				*APOB*	Up			
				*LRAT*	Up			
			Fat digestion and absorption	*FABP1*	Up	27.03	<0.0001	<0.001
				*AGPAT2*	Up			
				*APOA1*	Up			
				*MTTP*	Up			
				*APOA4*	Up			
				*PLA2G2F*	Down			
				*APOB*	Up			
				*NPC1L1*	Up			
				*GOT2*	Up			
				*MOGAT2*	Up			
		Endocrine system	PPAR signaling pathway	*FABP1*	Up	15.49	<0.0001	0.014
				*PCK1*	Up			
				*SCD*	Up			
				*ACAA1*	Up			
				*APOA1*	Up			
				*APOC3*	Up			
				*PLIN2*	Down			
				*SLC27A4*	Up			
				*PLIN1*	Down			
				*ACSL3*	Up			
				*ACSL5*	Up			
		Immune system	Hematopoietic cell lineage	*CD22*	Down	13.64	<0.0001	0.023
				*CD19*	Down			
				*CD3D*	Up			
				*IL1R2*	Down			
				*MS4A1*	Down			
				*TNF*	Down			
				*FCGR1A*	Down			
				*FCER2*	Down			
				*CD14*	Down			
				*CD1.1*	Down			
				*IL6R*	Down			
				*CD1C*	Down			
Liver	Metabolism	Amino acid metabolism	Arginine biosynthesis	*ARG2*	Up	15.79	0.0012	0.022
				*GPT2*	Up			
				*NAGS*	Up			

^1^ Values are expressed as the expression of genes in the feeding two times daily (M2) group/expression of genes in the feeding one time daily (M1) group. GO: gene ontology; KEGG: Kyoto Encyclopedia of Genes and Genomes.

**Table 5:** Validation of RNA sequencing results of samples in pigs feeding one time (M1) or twice daily (M2) by real-time quantitative PCR.

Gene ^3^	RNA Sequencing ^1^ (M1 vs. M2)		Real-time PCR ^2^ (M1 vs. M2)	
	*p* Value	Fold Change	*p* Value	Fold Change
*APOA1*	0.004	1.981	0.033	1.821
*APOA4*	<0.001	2.879	0.028	2.287
*APOC3*	<0.001	2.620	0.039	2.383
*NPC1L1*	0.041	1.545	0.045	1.954
*ACAA1*	0.013	1.549	0.042	1.319
*FABP1*	0.001	2.320	0.042	2.476
*PCK1*	0.020	1.733	0.044	1.612

^1^ Results based on RNA sequencing of six samples from two groups. ^2^ Results based on six individual samples from each group. ^3^ *APOA1: Apolipoprotein A1; APOA4: Apolipoprotein A4; APOC3: Apolipoprotein C3; NPC1L1: Niemann-Pick C1-Like 1; ACAA1: Acetyl-CoA acyltransferase 1; FABP1: fatty acid binding protein 1; PCK1: Phosphoenolpyruvate carboxykinase 1*.

## Appendix A

**Table A1:** Description of primers used in the RT-qPCR analysis of gene expression.

Items	Gene	Forward	Reverse
Target	*FABP1*	GGACATCGGAAATCGTGCAG	ACTGAACCACTGTCTTGACC
	*ACAA1*	CGAGCTTCTCTCTGCAGTCAT	TGGGATGTCACTCAGAAACTGG
	*NPC1L1*	CCCTCAGTTTTAGCATATGCAC	CTCCCACACTCATCGTAGAAAG
	*ABCC2*	AAAGGAGTGTTTGCGAAGAATC	TTTTAAGGACTTCAAACGCCTG
	*APOA1*	TTCAGGATGAAAGCCGTGGT	GTGGCAAAATCCTTCACCCG
	*APOA4*	GGAGGATCTCAGGCAGAAGC	CCTCCTCTTTGAGGGTGCTG
	*APOC3*	TCTGGACAAAATGCAGGACTAT	GTATAATCCCAGAAGTCGGTGA
	*PCK1*	CGATGTATGTCATCCCGTTCAG	TAACCAAGGGCTTTTTCAAAGG
Reference	*β-actin*	CCACGAAACTACCTTCAACTC	TGATCTCCTTCTGCATCCTGT

**Table A2:** Effects of feeding frequency on organ weight and their percentage in growing pigs ^1^.

Items	M1	M2	*p*-Value
Liver/kg	0.901 ± 0.016	0.939 ± 0.032	0.309
Kidney/kg	0.184 ± 0.013	0.195 ± 0.006	0.473
Spleen/kg	0.082 ± 0.005	0.081 ± 0.006	0.888
Stomach/kg	0.427 ± 0.046	0.471 ± 0.058	0.562
Small intestine/kg	1.752 ± 0.094	1.887 ± 0.170	0.503
Large intestine/kg	1.861 ± 0.137	1.897 ± 0.247	0.902
Liver: Body weight	0.0234 ± 0.0005	0.0242 ± 0.0005	0.310
Kidney: Body weight	0.0048 ± 0.0003	0.0050 ± 0.0002	0.410
Spleen: Body weight	0.0021 ± 0.0002	0.0021 ± 0.0002	0.826
Stomach: Body weight	0.011 ± 0.001	0.012 ± 0.001	0.524
Small intestine: Body weight	0.045 ± 0.002	0.049 ± 0.005	0.500
Large intestine: Body weight	0.048 ± 0.003	0.048 ± 0.005	0.986

M1, feeding one time daily; M2, feeding two times daily.

## References

[1] Schwarz N.A., Rigby B.R., La Bounty P., Shelmadine B., Bowden R.G. (2011). A review of weight control strategies and their effects on the regulation of hormonal balance. J. Nutr. Metab..

[2] Le Naou T., Le Floc’h N., Louveau I., van Milgen J., Gondret F. (2014). Meal frequency changes the basal and time-course profiles of plasma nutrient concentrations and affects feed efficiency in young growing pigs. J Anim. Sci..

[3] Schneider J.D., Tokach M.D., Goodband R.D., Nelssen J.L., Dritz S.S., DeRouchey J.M., Sulabo R.C. (2011). Effects of restricted feed intake on finishing pigs weighing between 68 and 114 kilograms fed twice or 6 times daily. J. Anim. Sci..

[4] Newman R.E., Downing J.A., Thomson P.C., Collins C.L., Henman D.J., Wilkinson S.J. (2014). Insulin secretion, body composition and pig performance are altered by feeding pattern. Anim. Prod. Sci..

[5] Colpoys J.D., Johnson A.K., Gabler N.K. (2016). Daily feeding regimen impacts pig growth and behavior. Physiol. Behav..

[6] Poulopoulou I., Eggemann A., Moors E., Lambertz C., Gauly M. (2018). Does feeding frequency during lactation affect sows’ body condition, reproduction and production performance?. Anim. Sci. J..

[7] Nazari S.A., Ganjkhanlou M., Bahnamiri H.Z. (2019). Effects of Omega-3 fatty acid supplement and feeding frequency on insulin sensitivity and carcass characteristics in Mahabadi goat kids. Small. Ruminant. Res..

[8] Liu J., Liu Z., Chen L., Zhang H. (2016). iTRAQ-based proteomic analysis reveals alterations in the liver induced by restricted meal frequency in a pig model. Nutrition.

[9] Liu J.B., Cai X., Xiong H., Zhang H.F. (2017). Effects of feeding frequency on meat quality traits and Longissimus muscle proteome in finishing pigs. J. Anim. Physiol. Anim. Nutr. (Berl)..

[10] Davidson A.J., Castañón-Cervantes O., Stephan F.K. (2004). Daily oscillations in liver function: Diurnal vs circadian rhythmicity. Liver Int..

[11] Dedourge-Geffard O., Palais F., Biagianti-Risbourg S., Geffard O., Geffard A. (2009). Effects of metals on feeding rate and digestive enzymes in Gammarus fossarum: An in situ experiment. Chemosphere.

[12] National Research Council (NRC) (2012). Nutrient Requirements of Swine.

[13] Su Y., Luo Y.H., Zhang L.L., Smidt H., Zhu W.Y. (2015). Responses in gut microbiota and fat metabolism to a halogenated methane analogue in S prague D awley rats. Microb. Biotechnol..

[14] Young M.D., Wakefield M.J., Smyth G.K., Oshlack A. (2010). Gene ontology analysis for RNA-seq: Accounting for selection bias. Genome. Biol..

[15] Mao X., Cai T., Olyarchuk J.G., Wei L. (2005). Automated genome annotation and pathway identification using the KEGG Orthology (KO) as a controlled vocabulary. Bioinformatics.

[16] Shannon P., Markiel A., Ozier O., Baliga N.S., Wang J.T., Ramage D., Amin N., Schwikowski B., Ideker T. (2003). Cytoscape: A software environment for integrated models of biomolecular interaction networks. Genome Res..

[17] Lotia S., Montojo J., Dong Y., Bader G.D., Pico A.R. (2013). Cytoscape app store. Bioinformatics.

[18] Hatori M., Vollmers C., Zarrinpar A., DiTacchio L., Bushong E.A., Gill S., Leblanc M., Chaix A., Joens M., Fitzpatrick J.A. (2012). Time-restricted feeding without reducing caloric intake prevents metabolic diseases in mice fed a high-fat diet. Cell. Metab..

[19] Chaix A., Zarrinpar A., Miu P., Panda S. (2014). Time-restricted feeding is a preventative and therapeutic intervention against diverse nutritional challenges. Cell. Metab..

[20] Rothschild J., Hoddy K.K., Jambazian P., Varady K.A. (2014). Time-restricted feeding and risk of metabolic disease: A review of human and animal studies. Nutr. Rev..

[21] Schneider J.D., Tokach M.D., Dritz S.S., Nelssen J.L., DeRouchey J.M., Goodband R.D. (2007). Effects of feeding schedule on body condition, aggressiveness, and reproductive failure in group-housed sows. J. Anim. Sci..

[22] Yan H., Cao S., Li Y., Zhang H., Liu J. (2019). Reduced meal frequency alleviates high-fat diet-induced lipid accumulation and inflammation in adipose tissue of pigs under the circumstance of fixed feed allowance. Eur. J. Nutr..

[23] Varbo A., Benn M., Tybjærg-Hansen A., Jørgensen A.B., Frikke-Schmidt R., Nordestgaard B.G. (2013). Remnant cholesterol as a causal risk factor for ischemic heart disease. J. Am. Coll. Cardiol..

[24] Telford D.E., Sutherland B.G., Edwards J.Y., Andrews J.D., Barrett P.H.R., Huff M.W. (2007). The molecular mechanisms underlying the reduction of LDL apoB-100 by ezetimibe plus simvastatin. J. Lipid. Res..

[25] Davis H.R., Hoos L.M., Tetzloff G., Maguire M., Zhu L.J., Graziano M.P., Altmann S.W. (2007). Deficiency of Niemann-Pick C1 Like 1 prevents atherosclerosis in ApoE−/− mice. Arterioscler. Thromb. Vasc. Biol..

[26] Jia L., Ma Y., Rong S., Betters J.L., Xie P., Chung S., Wang N., Tang W., Yu L. (2010). Niemann-Pick C1-Like 1 deletion in mice prevents high-fat diet-induced fatty liver by reducing lipogenesis. J. Lipid. Res..

[27] Temel R.E., Tang W., Ma Y., Rudel L.L., Willingham M.C., Ioannou Y.A., Davies J.P., Nilsson L.M., Yu L. (2007). Hepatic Niemann-Pick C1–like 1 regulates biliary cholesterol concentration and is a target of ezetimibe. J. Clin. Investig..

[28] Davis H.R., Zhu L.J., Hoos L.M., Tetzloff G., Maguire M., Liu J., Yao X., Iyer S.P., Lam M.H., Lund E.G. (2004). Niemann-Pick C1 Like 1 (NPC1L1) is the intestinal phytosterol and cholesterol transporter and a key modulator of whole-body cholesterol homeostasis. J. Biol. Chem..

[29] Davies J.P., Scott C., Oishi K., Liapis A., Ioannou Y.A. (2005). Inactivation of NPC1L1 causes multiple lipid transport defects and protects against diet-induced hypercholesterolemia. J. Biol. Chem..

[30] Chabowski A., Górski J., Luiken J.J., Glatz J.F., Bonen A. (2007). Evidence for concerted action of FAT/CD36 and FABPpm to increase fatty acid transport across the plasma membrane. Prostaglandins. Leukot. Essent. Fatty Acids.

[31] Lee R.G., Willingham M.C., Davis M.A., Skinner K.A., Rudel L.L. (2000). Differential expression of ACAT1 and ACAT2 among cells within liver, intestine, kidney, and adrenal of nonhuman primates. J. Lipid. Res..

[32] Nguyen T.M., Sawyer J.K., Kelley K.L., Davis M.A., Rudel L.L. (2012). Cholesterol esterification by ACAT2 is essential for efficient intestinal cholesterol absorption: Evidence from thoracic lymph duct cannulation. J. Lipid. Res..

[33] Young S.G., Cham C.M., Pitas R.E., Burri B.J., Connolly A., Flynn L., Pappu A.S., Wong J.S., Hamilton R.L., Farese R.V. (1995). A genetic model for absent chylomicron formation: Mice producing apolipoprotein B in the liver, but not in the intestine. J. Clin. Investig..

[34] van Greevenbroek M.M., Robertus-Teunissen M.G., Erkelens D.W., de Bruin T.W. (1998). Participation of the microsomal triglyceride transfer protein in lipoprotein assembly in Caco-2 cells: Interaction with saturated and unsaturated dietary fatty acids. J. Lipid. Res..

[35] Altmann S.W., Davis H.R., Zhu L.J., Yao X., Hoos L.M., Tetzloff G., Iyer S.P., Maguire M., Golovko A., Zeng M. (2004). Niemann-Pick C1 Like 1 protein is critical for intestinal cholesterol absorption. Science.

[36] Hall A.M., Smith A.J., Bernlohr D.A. (2003). Characterization of the acyl-CoA synthetase activity of purified murine fatty acid transport protein 1. J. Biol. Chem..

[37] Yan S., Yang X.F., Liu H.L., Fu N., Ouyang Y., Qing K. (2015). Long-chain acyl-CoA synthetase in fatty acid metabolism involved in liver and other diseases: An update. World. J. Gastroenterol..

[38] Cao A., Li H., Zhou Y., Wu M., Liu J. (2010). Long chain acyl-CoA synthetase-3 is a molecular target for peroxisome proliferator-activated receptor delta in HepG2 hepatoma cells. J. Biol. Chem..

[39] Kantor P.F., Lucien A., Kozak R., Lopaschuk G.D. (2000). The antianginal drug trimetazidine shifts cardiac energy metabolism from fatty acid oxidation to glucose oxidation by inhibiting mitochondrial long-chain 3-ketoacyl coenzyme A thiolase. Circ. Res..

[40] Xie W.D., Wang H., Zhang J.F., Li J.N., Can Y., Qing L., Kung H.F., Zhang Y.O. (2011). Enhanced peroxisomal beta-oxidation metabolism in visceral adipose tissues of high-fat diet-fed obesity-resistant C57BL/6 mice. Exp. Ther. Med..

[41] Krishnamoorthy S., Jin R., Cai Y., Maddipati K.R., Nie D., Pagès G., Tucker S.C., Honn K.V. (2010). 12-Lipoxygenase and the regulation of hypoxia-inducible factor in prostate cancer cells. Exp. Cell. Res..

[42] Schroeder F., Petrescu A.D., Huang H., Atshaves B.P., McIntosh A.L., Martin G.G., Hostetler H.A., Vespa A., Landrock D., Landrock K.K. (2008). Role of fatty acid binding proteins and long chain fatty acids in modulating nuclear receptors and gene transcription. Lipids.

[43] Atshaves B.P., Martin G.G., Hostetler H.A., McIntosh A.L., Kier A.B., Schroeder F. (2010). Liver fatty acid-binding protein and obesity. J. Nutr. Biochem..

[44] Huang H., McIntosh A.L., Martin G.G., Petrescu A.D., Landrock K.K., Landrock D., Kier A.B., Schroeder F. (2013). Inhibitors of fatty acid synthesis induce PPARα-regulated fatty acid β-oxidative genes: Synergistic roles of L-FABP and glucose. PPAR Res..

[45] Beylot M., Neggazi S., Hamlat N., Langlois D., Forcheron F. (2012). Perilipin 1 ablation in mice enhances lipid oxidation during exercise and does not impair exercise performance. Metabolism.

[46] Cerk I.K., Wechselberger L., Oberer M. (2018). Adipose triglyceride lipase regulation: An overview. Curr. Protein. Pept. Sci..

[47] Wu G., Borbolla A.G., Knabe D.A. (1994). The uptake of glutamine and release of arginine, citruline and proline by the small intestine of developing pigs. J. Nutr..

[48] Bredt D.S., Snyder S.H. (1994). Nitric oxide: A physiologic messenger molecule. Annu. Rev. Biochem..

